# Cerebral blood flow, blood supply, and cognition in Type 2 Diabetes Mellitus

**DOI:** 10.1038/s41598-016-0003-6

**Published:** 2016-12-05

**Authors:** Jacobus F. A. Jansen, Frank C. G. van Bussel, Harm J. van de Haar, Matthias J. P. van Osch, Paul A. M. Hofman, Martin P. J. van Boxtel, Robert J. van Oostenbrugge, Miranda T. Schram, Coen D. A. Stehouwer, Joachim E. Wildberger, Walter H. Backes

**Affiliations:** 1 Departments of Radiology & Nuclear Medicine, Maastricht University Medical Center, Maastricht, the Netherlands; 2 School for Mental Health and Neuroscience (MHeNS), Maastricht, the Netherlands; 3 Departments of Psychiatry and Neuropsychology, Maastricht University Medical Center, Maastricht, the Netherlands; 4 Department of Radiology, Leiden University Medical Center, Leiden, the Netherlands; 5 Cardiovascular Research Institute Maastricht (CARIM), Maastricht, the Netherlands; 6 Department of Neurology, Maastricht University Medical Center, Maastricht, the Netherlands; 7 Department of Internal Medicine, Maastricht University Medical Center, Maastricht, the Netherlands

**Keywords:** Diagnostic markers, Diabetes complications, Dementia

## Abstract

We investigated whether type 2 diabetes (T2DM) and the presence of cognitive impairment are associated with altered cerebral blood flow (CBF). Forty-one participants with and thirty-nine without T2DM underwent 3-Tesla MRI, including a quantitative technique measuring (macrovascular) blood flow in the internal carotid artery and an arterial spin labeling technique measuring (microvascular) perfusion in the grey matter (GM). Three analysis methods were used to quantify the CBF: a region of interest analysis, a voxel-based statistical parametric mapping technique, and a ‘distributed deviating voxels’ method. Participants with T2DM exhibited significantly more tissue with low CBF values in the cerebral cortex and the subcortical GM (3.8-fold increase). The latter was the only region where the hypoperfusion remained after correcting for atrophy, indicating that the effect of T2DM on CBF, independent of atrophy, is small. Subcortical CBF was associated with depression. No associations were observed for CBF in other regions with diabetes status, for carotid blood flow with diabetes status, or for CBF or flow in relation with cognitive function. To conclude, a novel method that tallies total ‘distributed deviating voxels’ demonstrates T2DM-associated hypoperfusion in the subcortical GM, not associated with cognitive performance. Whether a vascular mechanism underlies cognitive decrements remains inconclusive.

## Introduction

Type 2 diabetes mellitus (T2DM) is associated with cognitive decrements and an increased risk to develop dementia^1^. Furthermore, diabetes is related to complications related to damage of large blood vessels, including macrovascular disease such as coronary artery disease, peripheral vascular disease, and stroke^2^. In addition, many complications of diabetes due to impairment of small blood vessels arise, including neuropathy, nephropathy, and retinopathy^3^. In the brain, T2DM is associated with white matter hyperintensities (WMHs), often presumed to be of vascular origin^4^. Altered cerebral hemodynamics is one of the potential mechanisms thought to underlie the characteristic cognitive decrements^5,6^. Rather than studying WMHs, which are structural end-stage manifestations of impaired cerebral hemodynamics, it is also possible with advanced MRI techniques to investigate more functional or physiological cerebral characteristics, which may precede these structural changes. A prime candidate for this is actual cerebral blood flow (CBF), which can be measured noninvasively using arterial spin labeling, an MRI method that uses magnetically labeled arterial blood as a tracer^7^.

Several studies have attempted to relate T2DM with alterations in CBF, using a variety of techniques, study designs, and patient selection criteria, but results appear therefore not consistent as some report hypoperfusion, while others do not^8^. Most global CBF analysis methods either average over a volume to summarize the characteristics of that region of interest^9^, or assume a certain overlap of local perfusion abnormalities over subjects using voxel-based statistical parametric mapping techniques^10^. As the effect of T2DM on CBF is likely to be subtle, the former method might not be sensitive enough to detect changes, especially when relatively large regions are analyzed. The latter method assumes a regional anatomical overlap of tissue alterations, which might not be apt for a non-focal disease such as T2DM. The current study introduces an alternative method of analysis that aims to overcome these issues. In this method, the number of voxels that statistically deviate from a normative value are recorded as ‘distributed deviating voxels’ and their numbers are compared between groups. In addition to CBF, which is a local measure of tissue perfusion, it is also relevant to consider the functionality of the feeding arteries. Especially the (internal) carotid arteries are of interest, as these conduit arteries provide to a large extent the blood supply to the cerebrum.

We set out to address in a non-demented population of patients with T2DM, with a range in cognitive performance and healthy controls, whether T2DM and cognitive function are related to alterations in (macrovascular) blood flow in the internal carotid artery and (microvascular) perfusion in the cerebral grey matter (GM).

## Results

### Clinical characteristics

Table 1 shows the baseline characteristics of the low and high cognitive performance groups, as participants were selected based on cognitive status. The groups were matched for age, sex, education and T2DM status. Table 2 lists the clinical characteristics of the participants, based on T2DM status. T2DM was associated with higher fasting blood glucose levels, higher HbA1c levels, higher body mass index, higher diastolic as well as systolic blood pressure, more often hypertension, and higher WM (white matter) lesion loads (Table 2). With respect to cognition, T2DM participants scored significantly lower on baseline MMSE score (p = 0.006) compared with non-diabetes participants. Baseline MMSE did not differ from repeated MMSE (p = 0.30).

**Table 1:** Characteristics of the two cognition groups^a^.

	Lower cognition (n = 40)	Higher cognition (n = 40)	p-value
T2DM (%, n)	55.0 (n = 22)	47.5 (n = 19)	0.5^b^
Age (y)	61.1 ± 9.5	62.6 ± 6.6	0.4
Sex (male, %, n)	57.5 (n = 23)	55.0 (n = 22)	0.8^b^
Education			0.8^b^
Low (%, n)	15.0 (n = 6)	20.0 (n = 8)	
Middle (%, n)	47.5 (n = 19)	45.0 (n = 18)	
High (%, n)	37.5 (n = 15)	35.0 (n = 14)	
15-WLT total score	37.1 ± 10.0	49.8 ± 9.2	<0.001
Executive functioning (sec)	63.3 ± 35.2	34.8 ± 12.7	<0.001
Verbal fluency	20.3 ± 4.9	27.3 ± 5.5	<0.001
Cumulative cognition score	−2.30 ± 2.18	2.08 ± 1.28	<0.001

Data are mean ± standard deviation. T2DM, type 2 diabetes mellitus; WLT, (verbal memory) Word Learning Test.

^a^only participants who were included in the final analysis; Independent samples *t*-test;

^b^Pearson *χ* ^2^ test.

**Table 2:** Clinical characteristics of participants with and without T2DM.

	Participants with T2DM (n = 41)	Participants without T2DM (n = 39)	p-value
**T2DM-related variables**			
Duration of diabetes (years)	9.8 ± 6.7	—	
Fasting Blood Glucose (mmol/l)	7.5 ± 1.2	5.1 ± 0.3	<0.001
HbA1c (%)	6.7 ± 0.4	5.6 ± 0.4	<0.001
HbA1c (mmol/mol)	50.2 ± 4.9	38.0 ± 4.5	<0.001
*T2DM medication:*			
None (%)	12.2	100	<0.001^a^
Insulin (%)	2.4	—	
Oral medication (%)	75.6	—	
Insulin and oral medication (%)	9.8	—	
**Clinical variables**			
BMI (kg/m^2^)	29.2 ± 3.5	24.7 ± 2.8	<0.001
SBP (mmHg)	152 ± 18	131 ± 18	<0.001
DBP (mmHg)	83 ± 10	76 ± 13	0.013
Cardiovascular disease (%)	20.5	13.5	0.4
Hypertension (%)	95.1	38.5	<0.001^a^
Smoking status, never/former/current (%)	23.7/71.1/5.3	23.7/55.3/21.1	0.114^a^
**Cognitive Score**			
Cumulative cognition score	−0.60 ± 3.17	0.40 ± 2.36	0.117
Baseline MMSE total score	28.6 ± 1.4	29.4 ± 0.8	0.006

Data are mean ± standard deviation. T2DM, type 2 diabetes mellitus; HbA1c, glycated hemoglobin; BMI, body mass index, SBP, systolic blood pressure; DBP, diastolic blood pressure; MMSE, Mini-Mental State Examination. Independent samples *t*-test;

^a^Pearson *χ*^*2*^ test.

### Cerebral hemodynamics

The flow analysis revealed that the flow in the internal carotid arteries in T2DM (10.5 ± 2.2 cm^3^/s) was not significantly different from controls (10.8 ± 1.8 cm^3^/s, p = 0.6). For global measurements of GM CBF, significantly lower values were found in T2DM (28.3 ± 5.6 ml/100 g/min) compared with controls (31.5 ± 5.9 ml/100 g/min, p = 0.014). However, after including age and sex as covariates, the difference disappeared (p = 0.51), also after adding atrophy (p = 0.69) or carotid flow in a separate analysis (p = 0.52). Therefore, no post-hoc analyses for sub-regions were performed. Additionally, the statistical parametric mapping CBF technique also did not reveal any significant locally overlapping differences (both for FDR and regional FDR). The voxel based morphometry analysis of T1-weighted images revealed for T2DM a small region of increased atrophy in the left insular cortex.

### ‘Distributed deviating voxels’ method

**Figure 1:**
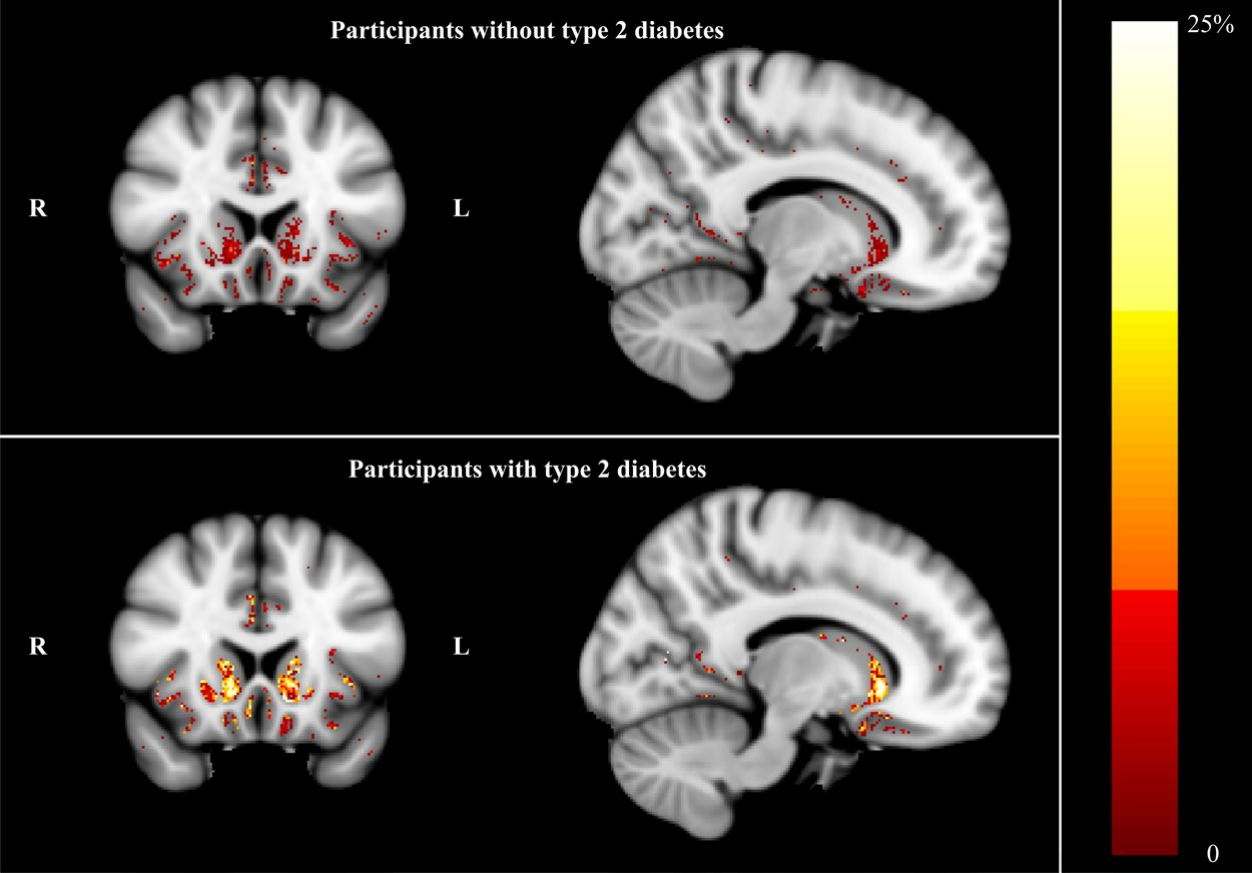
Normalized T1 weighted images, with as overlay the percentage of participants within a group displaying negatively ‘deviating voxels’ (using threshold Z < −2.576, indicative of hypoperfusion) for participants without T2DM (upper figure) and participants with T2DM (lower figure). Note the high percentage (indicating hypoperfusion) for T2DM in the subcortical GM, especially in the nucleus accumbens and caudate structures.

The ‘distributed deviating voxels’ method revealed approximately twice as many negatively deviating (low flow) voxels in the whole cerebrum for T2DM (0.10 ± 0.08% of intracranial volume (ICV)) compared with controls (0.05 ± 0.03% of ICV, p<0.001). Therefore post-hoc analyses were performed for the sub-regions (see Table 3). This analysis revealed that there were significantly more negatively deviating voxels for T2DM in frontal, temporal, parietal, and subcortical GM regions (p < 0.003), which only remained significant in the subcortical GM after correcting for atrophy (3.8-fold increase, p = 0.029, Fig. 1), also after correcting for carotid flow (p = 0.044). A similar trend was found for the frontal and temporal regions (p = 0.061 and p = 0.080, respectively). No regions were found showing more positively deviating (high flow) voxels for T2DM (2.27 ± 1.19% of ICV) compared with controls (2.56 ± 1.50% of ICV, p = 0.35). Age was a significant predictor of hypoperfusion (p = 0.041), but gender was not (p = 0.96).

**Table 3:** Fraction of negative ‘deviating voxels’ with low flow (hypoperfusion) in GM, relative to intracranial volume.

	Participants with T2DM (n = 41)	Participants without T2DM (n = 39)	p-value^a^
Cerebral cortex	0.10 ± 0.08%	0.05 ± 0.03%	<0.001^b^
Frontal cortex	0.04 ± 0.03%	0.02 ± 0.01%	0.003
Temporal cortex	0.03 ± 0.02%	0.01 ± 0.01%	<0.001
Occipital cortex	0.00 ± 0.00%	0.00 ± 0.00%	0.249
Parietal cortex	0.01 ± 0.01%	0.00 ± 0.00%	<0.001
Subcortical GM	0.02 ± 0.02%	0.01 ± 0.01%	<0.001^b^

Data are mean  ± standard deviation. GM, grey matter; T2DM, type 2 diabetes mellitus.

^a^Independent samples *t*-test,

^b^Significant after correcting for age, sex, and atrophy.

Post-hoc regression analyses revealed that the total of negative deviating voxels in subcortical GM was (positively) associated with self-reported diabetes duration (beta = 0.406, p = 0.005), but that none of the cardiovascular or glycemic measures were significant predictors (p > 0.19). However, the post-hoc analysis did reveal that some measures did have an effect on the group difference (by including it as covariate, the group difference disappeared: fasting blood glucose (p = 0.47), BMI (p = 0.20), presence of hypertension (p = 0.09), self-reported diabetes duration (p = 0.97)), whereas for other measures the significant group difference remained intact (HbA1c (p = 0.040), systolic blood pressure (p = 0.045), cardiovascular disease (p = 0.032), diastolic blood pressure (p = 0.032)).

Furthermore, linear regression, with inclusion of cognitive performance as covariate, also did not yield any significant association between cognition with flow or CBF (p > 0.37). Association with depression was not significant for the region of interest analysis (p > 0.11), but a significant (positive) association was found when included in the deviating voxels analysis, particularly for the whole brain analyses, and the individual frontal, temporal and subcortical grey matter (p < 0.003).

## Discussion

This study was performed to investigate whether T2DM and cognitive impairment are associated with differences in cerebral blood flow. To this end, participants with T2DM, with a range in cognitive performance and healthy controls, were investigated by use of MRI flow techniques: a macrovascular flow technique to study the blood supply from the internal carotid artery, and an arterial spin labeling technique to measure the microvascular grey GM perfusion in terms of CBF. The distributed deviating voxels method revealed that participants with T2DM exhibited significantly more GM tissue with low CBF values in the cerebral cortex and, particularly, the subcortical GM. Interestingly, depression was associated with low CBF values in the subcortical GM. No (independent) associations were found between carotid artery or GM blood flow and cognitive decrements.

T2DM was not associated with carotid artery blood flow, which is fully in agreement with previous studies^11,12^, and indicates that blood supply to the brain is not affected in the investigated T2DM population. However, the current study did observe more GM tissue with abnormally low flow values in the cortex and the subcortical GM in T2DM, which can be interpreted as evidence for cerebral hypoperfusion. The fact that age was also a significant predictor of hypoperfusion, fits the theory that T2DM accelerates ageing^13^. This finding concurs with the hypothesis that T2DM is associated with impaired cerebral hemodynamics, a mechanism that might in part underlie the cognitive decrements or accelerated cognitive aging associated with T2DM^5^. It has been suggested that T2DM can affect the glucose and insulin transfer across the blood-brain barrier, hence altering regional metabolism and microcirculation^14^. Chronic hyperglycemia has been shown to decrease regional blood flow and increase membrane permeability, eventually prompting permanent brain cell damage^15^. A progressive metabolic disturbance in the cerebrovascular bed seems to disturb blood flow and accelerate WM degeneration^14^. Interestingly, hypertension was not associated with CBF, which might indicate that the cerebral autoregulation is still intact in this relatively healthy population.

An explanation why the CBF association was detected in the subcortical GM, but not in the cortical regions, might be a different local microvascular architecture: vessel density is lower and vessels are more deep than collateral in the subcortical region compared with the cortical region^16^. Furthermore, in small vessel disease, for which T2DM is a risk factor, microbleeds are more often found subcortical^17^, indicating that the subcortical region might have a higher susceptibility to vascular pathology (e.g. ischemia or hypoperfusion). Furthermore, we observed an association of depression with subcortical CBF measures. It has previously been shown that major depressive disorder is associated with structural subcortical alterations^18^, therefore a compromise of the blood supply to the subcortex and its connections can result in behavioral syndromes, including depression^19^.

Over the last 2 decades, the association of T2DM with CBF has been investigated in a number of studies, using various techniques including single-photon emission computed tomography, positron emission tomography and ASL. Although some report hypoperfusion in T2DM^10,14,20,21^, other studies did not find any association with CBF^11,22,23^. It has been shown that most studies reporting on hypoperfusion in T2DM typically use small populations, include patients with severe complications, and did not account for atrophy, which has been shown to largely explain hypoperfusion^24^. The latter notion was also evidenced in the current study, as including atrophy as covariate decreased the number of regions with hypoperfusion, but the results in subcortical GM remained significant. Hence, the effect of T2DM on CBF, independent of atrophy, is small. Furthermore, a regional analysis of atrophy (voxel based morphometry) did not indicate atrophy in the subcortical GM, providing more support that the hypoperfusion in the subcortical GM is indeed independent of atrophy.

Larger, epidemiologic studies have failed so far to find associations of CBF with T2DM^9,25^. Tiehuis *et al*. suggested that despite the absence of an association of T2DM with CBF under resting conditions, it is still possible that T2DM is associated with altered cerebrovascular reactivity^11^. Indeed, research specifically designed to assess cerebral vasoreactivity using ASL under hypercapnic conditions reported that patients with T2DM exhibit diminished global and regional cerebral vasoreactivity^23,26^. In addition, the work from Duarte *et al*., who used tasked-based functional MRI, showed that T2DM is associated with impaired neurovascular coupling, as the hemodynamic response function is different from healthy controls^27^.

A possible explanation for the fact that the current study does find evidence of cerebral hypoperfusion under resting conditions, even when correcting for atrophy, might be attributable to the potentially higher sensitivity of the analysis applied. The current study applied three distinctly different methods to assess the effect of T2DM or cognitive status on CBF. The applied methods were sensitive to either 1) global effects (region of interest analysis), 2) locally overlapping focal effects (voxel-based statistical parametric mapping), or 3) more spatially distributed (diffuse) effects (‘deviating voxels’). Only the deviating voxel method appeared sensitive enough to detect a significant effect of T2DM on CBF.

An explanation for this is that the CBF effect of T2DM is probable subtle (which is reflected by the very low percentage of deviating voxels), and not necessarily localized at identical spots across different individuals. These effects might not be picked up by a region of interest analysis, as subtle effects in sub regions might be overshadowed by noise from other sub regions when taking an average over a selection of sub regions. Furthermore, when a large number of regions is considered, one has to correct for multiple comparisons, thereby decreasing the likelihood of obtaining significant effects. Additionally, voxel-based statistical parametric mapping technique could be insensitive to such effects, as this method assumes a certain regional overlap across individuals of altered tissue. In contrast with more focal pathologies such as epilepsy and stroke, T2DM is a systemic disease, and although there might be regional differences, there is no evidence that these regions should be identical for different patients with T2DM. The current study introduced an alternative method of analyzing (‘distributed deviating voxels’) that proved to be more sensitive than the other two analysis techniques. By tallying the number of deviating voxels, changes in CBF can be detected that are subtle while not requiring overlap at the exact location of the hemodynamic disturbance over the studied participants.

In contrast with other studies on blood flow in T2DM^5,11,22,23,25^, no significant associations of cognitive performance status were found with CBF. In the current, non-demented population of participants with T2DM, the cognitive performance for all participants either with or without T2DM falls within the range considered cognitively normal (i.e. MMSE > 27). The individuals that do experience cognitive decrements still exhibit substantially better cognitive performance scores than for example patients who are suspect of a cognitive disorder (MMSE < 25). A potential implication of this study that found a significant association between CBF and T2DM status, but not between CBF and cognitive performance, is that T2DM-induced cerebrovascular alterations potentially precede the cognitive decrements. Therefore, an altered CBF might be a potential biomarker to identify patients at risk of developing cognitive problems. Interestingly, we did find a positive association of depression with subcortical CBF (deviating voxel analysis), which fits the theory of a vascular pathology underlying depression^19^. A post-hoc analysis showed that none of the cardiovascular or glycemic measures were significant predictors for subcortical CBF, however some measures, including fasting blood glucose, BMI, and presence of hypertension were shown to affect the group difference, which hints at some sort of cardiovascular and glycemic mechanisms underlying hypoperfusion in T2DM.

T2DM was found to be associated with hypoperfusion, hence the results of this study indicate that treatment to avoid decline of or even improve vascular function (e.g. with antihypertensive or antiplatelet drugs) could be beneficial in patients with T2DM. Future studies that further elucidate these biological alternations might reveal new opportunities to monitor therapeutic/lifestyle interventions for improving cognition and/or prevention of cognitive impairment. A longitudinal set up is required to investigate whether T2DM patients with cerebral hypoperfusion are at increased risk for developing cognitive decrements in the near future.

The strengths of the present study comprise: first, the extensive (cardiovascular) characterization of the participants. Second, the investigation included both microvascular GM perfusion and macrovascular blood supply, and incorporated a variety of CBF analysis methods. Third, the quantified measures for carotid flow (approximately 11 cm^3^/s) and CBF (approximately 32 ml/100 g/min) are comparable with previously reported values of 8 cm^3^/s for carotid flow^28^ and 30 ± 5 ml/100 g/min ([^15^O]H_2_O PET) and 34 ± 5 ml/100 g/min (ASL) for CBF^29^, which is indicative of sound quantitative results. On the other hand, limitations should also be considered. A first limitation is the cross-sectional design of the study. Nevertheless, these first cross-sectional results are promising and pave the way for future (longitudinal) studies. Second, the inclusion of relative healthy subjects with T2DM decreased the likelihood of finding a possible association between cognition and CBF, as observed in other studies, but might provide a more representative view of early effects of diabetes on cognition. The subjects with TD2M included in our study were selected from a community-dwelling population and are in excellent control, therefore our results only apply to T2DM with outstanding control. A post-hoc analysis with diabetes duration as measure for ‘severity’ did show a positive relationship between hypoperfusion and ‘severity’. However, this analysis is not entirely reliable, as only 39 subjects were included, and duration as a measure for diabetes severity is typically a self-reported, and therefore imprecise measure, and its misclassification may obscure analysis^30^. Nonetheless, it will be interesting to include more severe diabetes participants in the future. Lastly, for the deviating voxel analysis, we used the non-diabetic, high cognitive performers as reference group, which might not be ideal as this group was not free of smokers or hypertension. However, we are confident that our results are sound, as we already find differences with respect to this group, and we would expect to only find stronger effects if we had the opportunity to select a ‘cleaner’ reference group.

To conclude, a novel analysis method that tallies total ‘deviating voxels’ demonstrates distributed hypoperfusion in T2DM, especially in the subcortical regions, whereas more traditional analysis methods appeared to be not sensitive enough. Whether a vascular mechanism underlies the cognitive decrements in T2DM remains inconclusive.

## Methods

### Study population

Forty-seven participants with T2DM and forty-one participants without T2DM mellitus were recruited from the first 866 participants in the community-dwelling population of the Maastricht Study^31^ for additional brain MRI measurements. Participants were considered to have diabetes according to the WHO 2006 criteria if they used diabetes medication, or if they had a fasting blood glucose ≥7.0 mmol/L, and/or a 2-hour blood glucose ≥11.1 mmol/L after an oral glucose tolerance test. Participants without T2DM were characterized by fasting blood glucose <6.1 mmol/L and a 2-hour blood glucose <7.8 mmol/L. At baseline inclusion, participants underwent an extensive battery of measurements, including cognitive performance tasks, blood pressure measurements, and blood sampling^31^. After these measurements, participants were invited to participate in this additional MRI examination.

Participants with the highest and lowest cognitive scores were selected from the first 866 participants to obtain a range in cognitive scores (Table 1), as has been described previously^32^. The division of participants into a low and high cognition group was based on a cumulative score of three neuropsychological tests covering the domains of verbal memory^33^, attention and flexibility, and executive functioning^34^ and verbal fluency^35^ (Table 1). This selection was performed to increase the possibility of finding cerebral differences, as the effect of T2DM on cognition is only mild in non-demented subjects^36^. Scores were adjusted for age, sex, and education level using linear regression analysis. The low and high cognition groups were matched for age, sex, and education level, and displayed a comparable number of participants with and without T2DM (Table 1), for demographic characteristics based on diabetes status, please see Table S1 in the Supplementary Information. Depression was assessed using the Mini International Neuropsychiatric Interview (MINI)^37^.

A total of 41 and 39 participants with and without T2DM with reliable data were included, respectively. The study was approved by the Medical Ethics Committee of the Maastricht University Medical Center (MUMC+), the Netherlands, and all participants gave written informed consent. Furthermore, all methods described in this manuscript were carried out in accordance with the approved guidelines. The study was registered at http://www.clinicaltrials.gov with identifier NCT01705210.

### Magnetic resonance imaging

MRI data were acquired on a 3T scanner (Achieva TX, Philips Healthcare, Best, the Netherlands) using a 32-element head coil for parallel imaging. The MRI protocol consisted of structural scans (including T1-, T2-, T2*-weighted and fluid attenuated inversion recovery sequences), phase-contrast angiograms, quantitative flow of the carotid artery, and whole cerebrum arterial spin labeling. A three-dimensional T1-weigthed (T1) fast field echo sequence (TR/TE 8.1/3.7 ms, 8° flip angle, 1 mm isotropic voxel size, 170 continuous slices, matrix size of 240 × 240) was used as anatomical reference.

**Figure 2:**
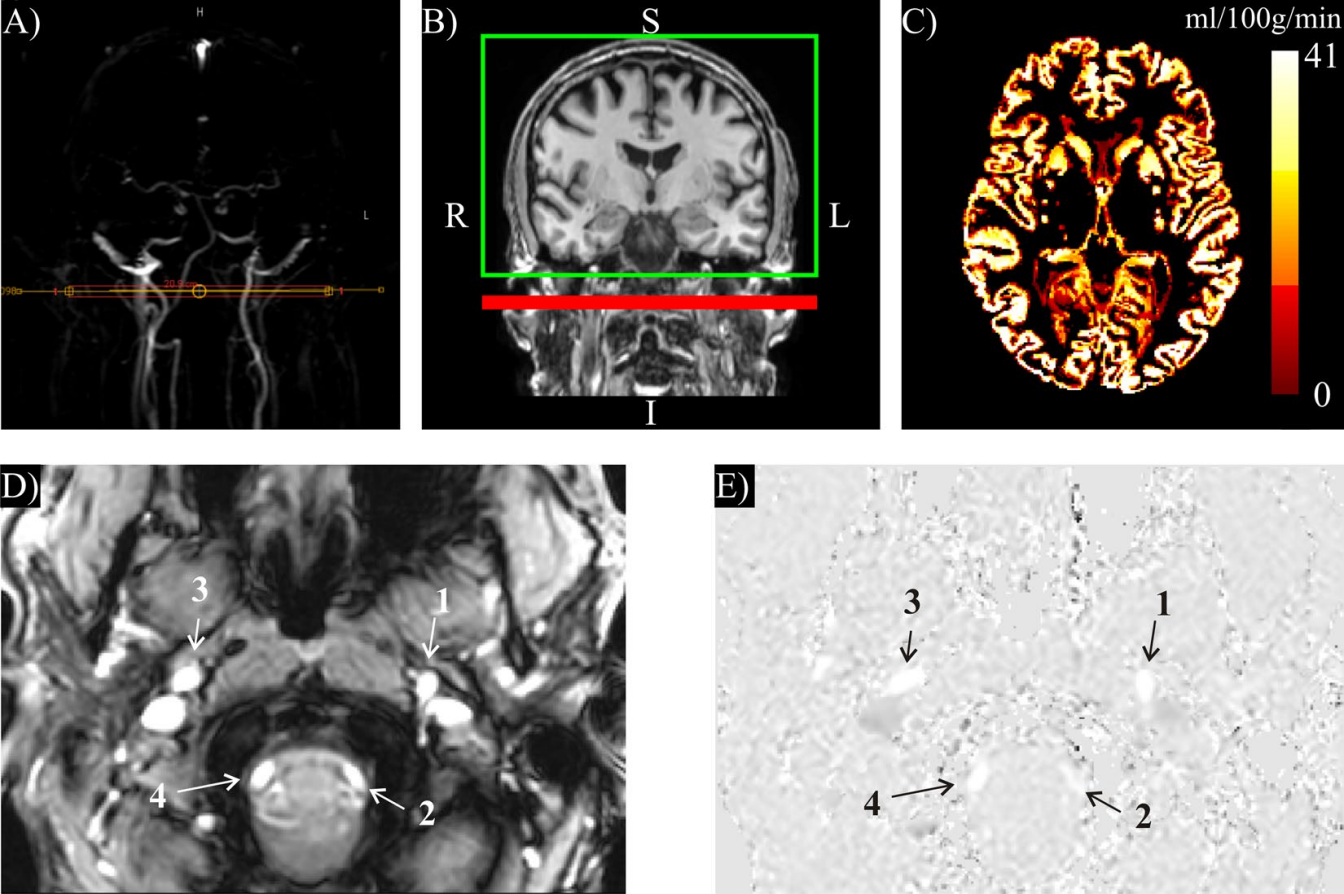
(**A**) Coronal maximum intensity projections derived from phase-contrast angiography with indication of the slice for quantitative flow measurement in the internal carotid artery in a participant with T2DM. (**B**) Sagittal T1 weighted image with labeling slice (red), which was positioned at the same location of the slice for quantitative flow estimation, and imaging volume (green). (**C**) Resulting transverse CBF map. (**D**) Magnitude and (**E**) phase images of the carotid region, 1 left internal carotid artery, 2 left vertebral artery, 3 right internal carotid artery, 4 right vertebral artery.

Vascular anatomy from the common carotid artery to a level distal to the circle of Willis was determined using three-dimensional phase-contrast MR angiography. Maximum intensity projections in orthogonal directions resulted in three-dimensional angiograms which were used to position the two-dimensional slice for the quantitative flow estimation (Q-flow, Philips Medical Systems) (Fig. 2A) and the labeling slice for quantitative CBF estimation (Fig. 2B).

The Q-flow technique was based on a single-slice, multiphase, fast-field echo sequence which encoded velocities parallel to the slice-encode direction. The slice was placed perpendicular to the internal carotid artery, distal to the bifurcation, on a position where the artery appeared least tortuous. Measurements were made in both the left and right sides of the carotid arteries, and were subsequently averaged over the entire cardiac cycle (Fig. 2D). Carotid flow images were acquired using 2D fast cine PC-MRI pulse sequence with retrospective ECG gating with 15 time frames covering the entire cardiac cycle. Phase-contrast parameters were as follows: TE/TR 8/13 ms, flip angle 10°, field-of-view 150 × 105 mm^2^, matrix 128 × 88, and slice thickness 6 mm. Flow direction: craniocaudal, encoding velocity: 120 cm/s.

Subsequently, a pseudo-continuous (PC) ASL 2D multislice single-shot echo planar imaging (EPI) sequence^38^ was acquired with a TR/TE of 3847/14 ms, voxel size of 3 × 3 × 7 mm^3^, matrix size of 80 × 80 × 17, a post labeling delay (PLD) of 1525 ms for the first slice (PLD of last slice was 2085 ms) and a label duration of 1650 ms. The labeling slice was positioned at the same location as for the Q-flow technique (Fig. 2B). Slices were obtained in the feet-head direction (35 ms per slice), and 50 control-tag pairs were acquired. A single proton density (PD) sequence was acquired with a TR of 10 s to scale the PCASL signal intensity to an absolute CBF value.

### Data analysis

The T1 weighted images were automatically segmented to obtain total intracranial volume (ICV), and lateral ventricle size using the Freesurfer software package (Martinos Center for Biomedical Imaging, Boston, USA)^39^. The total lateral ventricular volume was taken as a measure for atrophy.

Carotid flow images were analyzed in Matlab. Arteries were visually identified and delineated on the magnitude image to segment the flow regions on the phase images for all time frames. Flow was calculated in cm^3^/s by integrating the velocity value over the pixels of the vessel cross-section and averaging over the time frames. Due to technical difficulties, not all flow measurements were successful. For the final flow analysis, reliable data were available in 36 (of 47) participants with T2DM and 37 (of 41) healthy controls.

For ASL, motion correction was performed relative to the mean of the control images with FMRIB’s linear image registration tool (FLIRT) using a mutual-information algorithm^40^. Next, the label images were subtracted from the control images. Control-tag pairs were removed when visual inspection of the subtraction result showed abnormal results. CBF maps calculation (Fig. 2C) was based on the ASL whitepaper^41^ with correction for a 2D multislice acquisition scheme and a correction factor for background suppression^42^, see equation 1:

1 $${\rm{CBF}}=\frac{6000\cdot \lambda \cdot ({{\rm{SI}}}_{{\rm{control}}}-{{\rm{SI}}}_{{\rm{label}}})\cdot {e}^{\frac{{T}_{{\rm{delay}}}+{T}_{{\rm{slice}}}(z-1)}{{T}_{1,{\rm{blood}}}}}}{2\cdot \alpha \cdot {\alpha }_{{\rm{inv}}}\cdot {T}_{1,{\rm{blood}}}\cdot {{\rm{SI}}}_{{\rm{PD}}}\cdot (1-{e}^{\frac{-\tau }{{T}_{1,{\rm{blood}}}}})}$$

where λ is the blood-brain partition coefficient (set at 0.9 ml/g), SI_control_ and SI_label_ are the means over time of the control and label images, respectively, *T*_delay_ is the post label delay (1525 ms), *T*_slice_ is the acquisition time for a single slice (35 ms), *z* is the slice number, *T*_*1*,blood_ is the longitudinal relaxation time of blood (set at 1650 ms for 3T), α is the labeling efficiency (set at 0.85), α_inv_ is a correction factor for the background suppression (set at 0.83), SI_PD_ is the signal intensity of the proton density image and *τ* is the label duration (1650 ms). A GM probability map was created from the T1-weighted structural scan using FAST^43^, which allowed for correction of the CBF for the amount of GM in a voxel^44^. Finally, CBF maps were coregistered to Montreal Neurological Institute space using FNIRT^45^ to use the MINC1 atlas^46^.

For the regional analysis, CBF values were expressed in ml/100 g/min and averaged over the GM of the following regions: whole cerebral cortex, frontal, temporal, parietal, and occipital cortex, and subcortical GM (i.e. accumbens, caudate, pallidum, putamen, and thalamus), as defined by the MINC1 atlas^46^.

Additionally, a voxel-based statistical parametric mapping analysis was performed on CBF maps (to assess regional anatomical overlap of altered CBF values) and separately on T1-weighted images (voxel based morphometry, to assess overlapping regions with atrophy) using routines from the SPM8 software package (Wellcome Department of Cognitive Neurology). Age and sex were added as covariates, and correction for multiple comparisons was applied using a False Discovery Rate (FDR) of 5%. To increase the sensitivity by exploiting the spatially clustered nature of effects, an additional analysis was performed using a regional FDR^47^ of 5%.

Finally, for the ‘distributed deviating voxels’ analysis, the CBF maps of the subjects were transformed on a pixel-by-pixel basis into a statistical *z*-score (defined as [(x_i_ − x_ref_)/SD_ref_]) maps using the locally averaged CBF values of the controls, with the highest cognitive performance (x_ref_) and its standard deviation (SD_ref_), as reference 48,see Table S2 in the Supplemental Information for their summarizing characteristics. The *z*-score maps of the participants with the highest cognitive performance within the control group were based on the values of the other high cognitive performance controls (n-1). (n-1) stands for total of subjects within the reference group minus that specific individual (using the one leave out method). For all regions (whole brain, frontal, temporal, parietal, and occipital cortex, and subcortical GM, as defined by the MINC1 atlas), the voxels were counted that deviated with 99% confidence, corresponding to *z*-score of z_α/2_  =  2.576. Both positive and negative *z*-values were considered separately. The number of voxels are reported as percentage of the total intracranial volume.

For the CBF analysis, 6 participants with T2DM were excluded due to claustrophobia (n = 2), Parkinsonism (n = 1), brain injury because of an accident (n = 1), an incidental finding (n = 1) and a major artifact in the ASL data (n = 1). For the participants without T2DM, 2 excluded due to non-diabetes participants with impaired fasting blood glucose levels (n = 2). For carotid flow, an additional 5 subjects with T2DM and 2 without T2DM were excluded based on incomplete Qflow data due to no ECG signal.

Therefore, reliable data were available in 41 (of 47) participants with T2DM and 39 (of 41) healthy controls.

### Statistical analysis

Descriptive participants’ characteristics are reported as mean ± standard deviation. Group characteristics were tested by use of independent samples *t*-tests and Pearson *χ* ^*2*^-tests with SPSS (Statistical Package for Social Sciences, version 20, IBM Corp., USA), with α = 0.05.

Differences in carotid flow and CBF measures between T2DM and controls were tested by use of independent samples *t*-tests. When differences were significant, they were subsequently explored with linear regression analysis, to correct for differences in clinical characteristics between groups. For carotid flow, the linear regression analysis was adjusted for age and sex. For CBF measures (global GM CBF, and number of deviating CBF voxels relative to ICV), first age and sex were used as covariates in the analysis. Subsequently, lateral ventricular volume (as measure for atrophy) and carotid flow were separately added as covariates. Atrophy was included, as it is known to affect CBF^24^. Subsequently, the association with and the effect on CBF of cardiovascular, glycemic measures, and self-reported diabetes duration was explored in a post-hoc fashion by adding these measures separately as covariates to the regression model which included age, sex, and atrophy.

Furthermore, to limit the number of statistical tests for the CBF analyses, in a staged approach, first only the whole cerebrum was considered. When significant differences were observed for the whole cerebrum, exploratory post-hoc tests were subsequently performed to evaluate the sub-regions (frontal, temporal, parietal, and occipital cortex, and subcortical GM). Finally, to evaluate the association of cognitive performance or depression, a dichotomous value (low versus high cognition, or presence of major depressive episode, respectively) was added to the linear regression models for flow and CBF.

## Electronic supplementary material

Supplementary Information
