# Supplementary material for: Cerebral blood flow, blood supply, and cognition in Type 2 Diabetes Mellitus
Source: Sci Rep. 2016 Dec 5;6:160003. doi: 10.1038/s41598-016-0003-6 (PMC8276879; doi:10.1038/s41598-016-0003-6)
Supplement: Supplementary file 1 — Supplementary Information [file 41598_2016_3_MOESM1_ESM.doc]

**Supplementary Information:**

**Cerebral blood flow, blood supply, and cognition in Type 2 Diabetes Mellitus**

Jacobus F.A. Jansen^a,b,*^, Frank C.G. van Bussel^a,b,*^, Harm J. van de Haar^a,b,d^, Matthias J.P. van Osch^g^, Paul A.M. Hofman^a,b^, Martin P.J. van Boxtel^b,d^, Robert J. van Oostenbrugge^b,c,e^, Miranda T. Schram^c,f^, Coen D.A. Stehouwer^c,f^, Joachim E. Wildberger^a,c^, and Walter H. Backes^a,b^

**Table S1. Characteristics of the groups based on T2DM status**

|  | T2DM | Controls |
| --- | --- | --- |
| Age (years) | 64.7 ± 6.2 | 58.8 ± 9.0^a^ |
| Sex, male (%) | 73.2 | 38.5^b^ |
| Education, low/middle/high (%) | 19.5 / 58.5 / 22.0 | 15.4 / 33.3 / 51.3^b^ |
| Data are mean ± SD. ^a^ P < 0.01, independent samples Student *t*-test. ^b^ P < 0.01 Pearson χ2‐test | | |

**Table S2. Characteristics of the reference group: controls with the highest cognitive performance**

|  | Control cogn high Group (n=21) |
| --- | --- |
| Age (years) | 60.7 ± 7.7 |
| Sex, male (%) | 28.6 |
| Education, low/middle/high (%) | 19.0 / 33.3 / 47.6 |
| MMSE | 29.7 ± 0.5 |
| 15-WLT total score | 53.2 ± 7.3 |
| Executive functioning (sec) | 31.8 ± 11.8 |
| Verbal fluency | 29.1 ± 6.3 |
| Cumulative cognition score | 2.31 ± 1.28 |
| Fasting Blood Glucose (mmol/l) | 5.0 ± 0.3 |
| HbA_1c_ (%) | 5.7 ± 0.3 |
| HbA_1c_ (mmol/mol) | 39.3 ± 3.7 |
| BMI (kg/m^2^) | 24.2 ± 3.0 |
| SBP (mmHg) | 132 ± 20 |
| DBP (mmHg) | 76 ± 12 |
| Cardiovascular disease (%) | 15.0 |
| Hypertension (%) | 47.6 |
| Smoking status, never/former/current (%) | 19.0 / 57.1 / 23.8 |
| Data are mean ± SD. MMSE, Mini-Mental State Examination; WLT, (verbal  memory) Word Learning Test; HbA_1c_, glycated hemoglobin; BMI, body mass index, SBP, systolic blood pressure; DBP, diastolic blood pressure | |
